# Supplementary figures and images for: Antiviral activity of intracellular nanobodies targeting the influenza virus RNA-polymerase core
Source: PLoS Pathog. 2024 Jun 14;20(6):e1011642. doi: 10.1371/journal.ppat.1011642 (PMC11210859; doi:10.1371/journal.ppat.1011642)

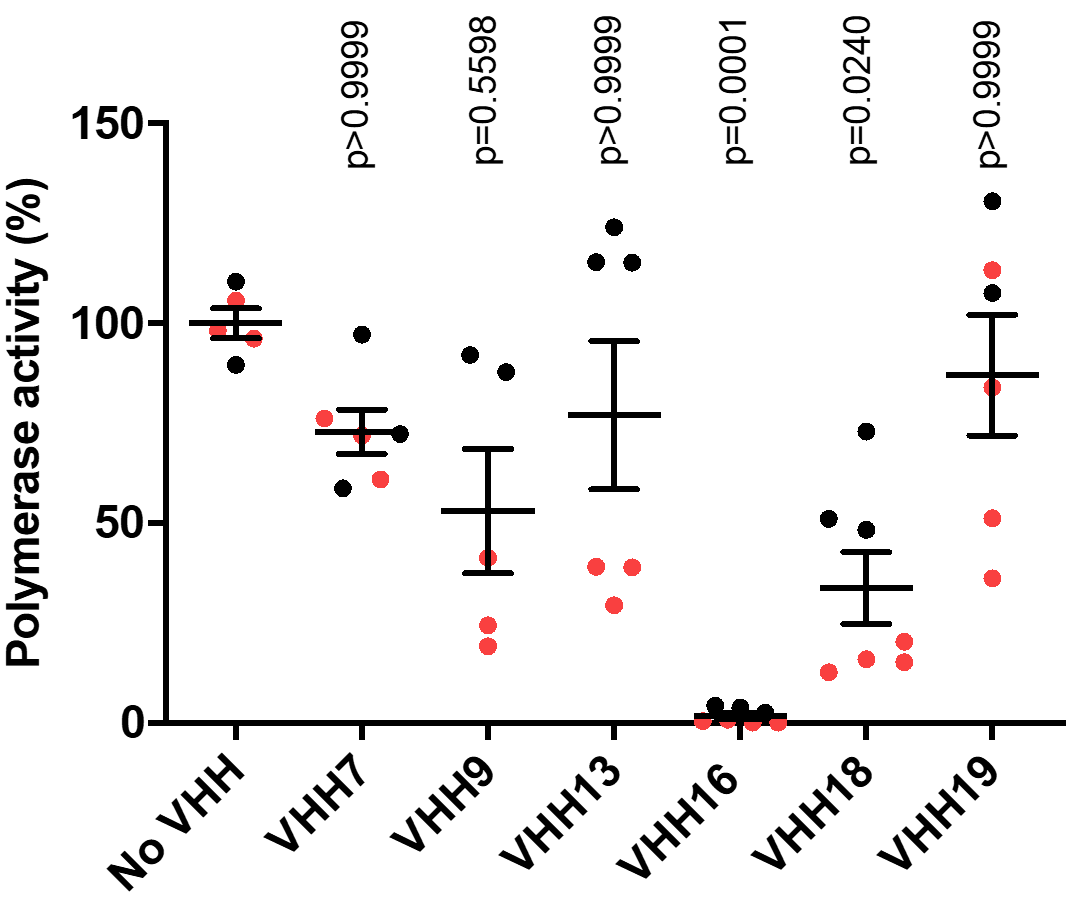

Supplement: S1 Fig — Plasmids expressing NP, PA, PB1, PB2 of the WSN (H1N1) strain were co-transfected in HEK-293T cells together with the NA-firefly-luciferase reporter plasmid and a plasmid encoding a VHH or an empty plasmid (indicated as No VHH). A plasmid encoding the nano-luciferase was co-transfected to control DNA uptake and normalize minireplicon activity. Luciferase activities were measured in cell lysates 48 hours post-transfection. Colors indicate distinct biological replicates. (TIF) [file ppat.1011642.s001.TIF]

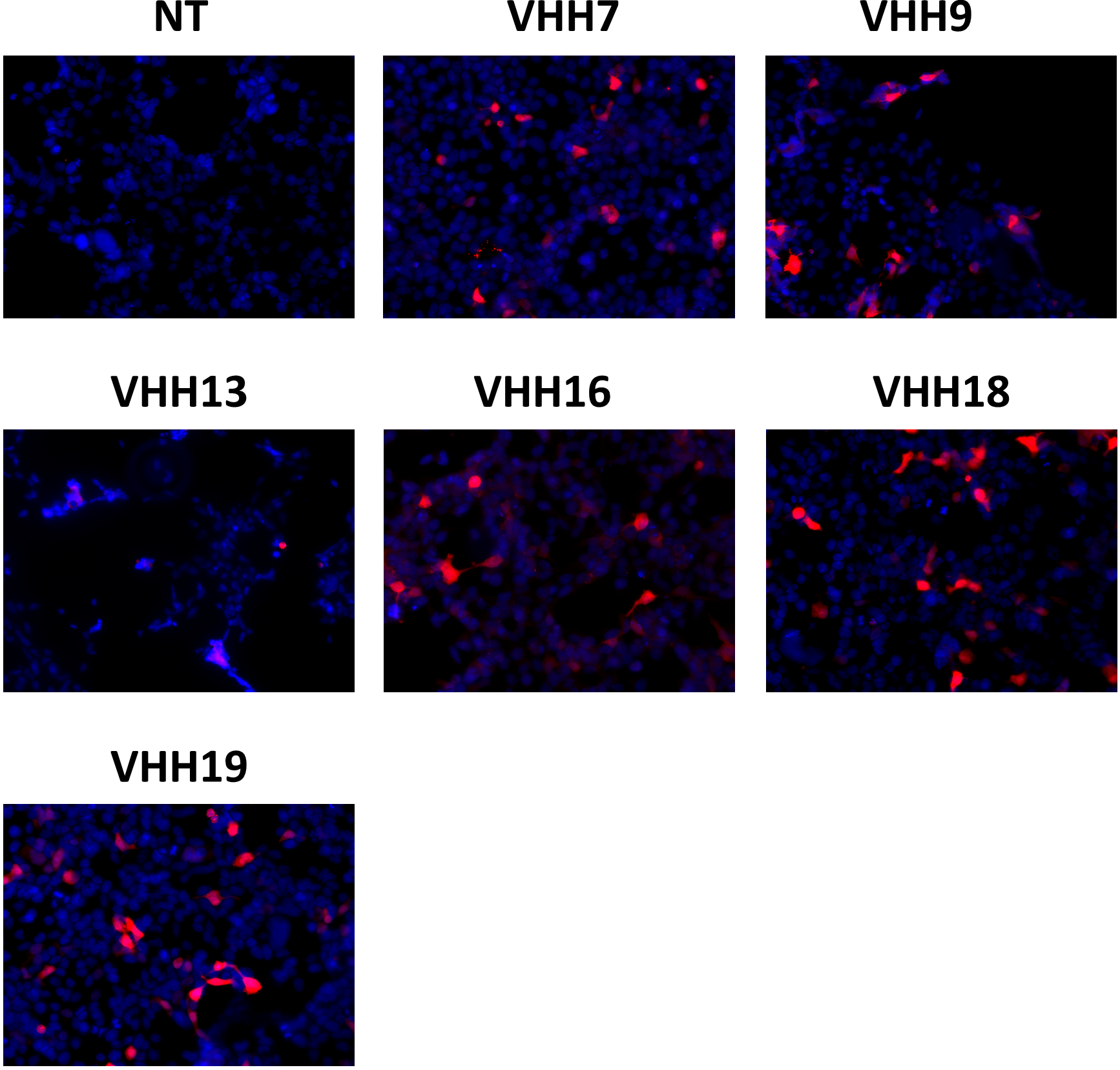

Supplement: S2 Fig — (TIF) [file ppat.1011642.s002.TIF]

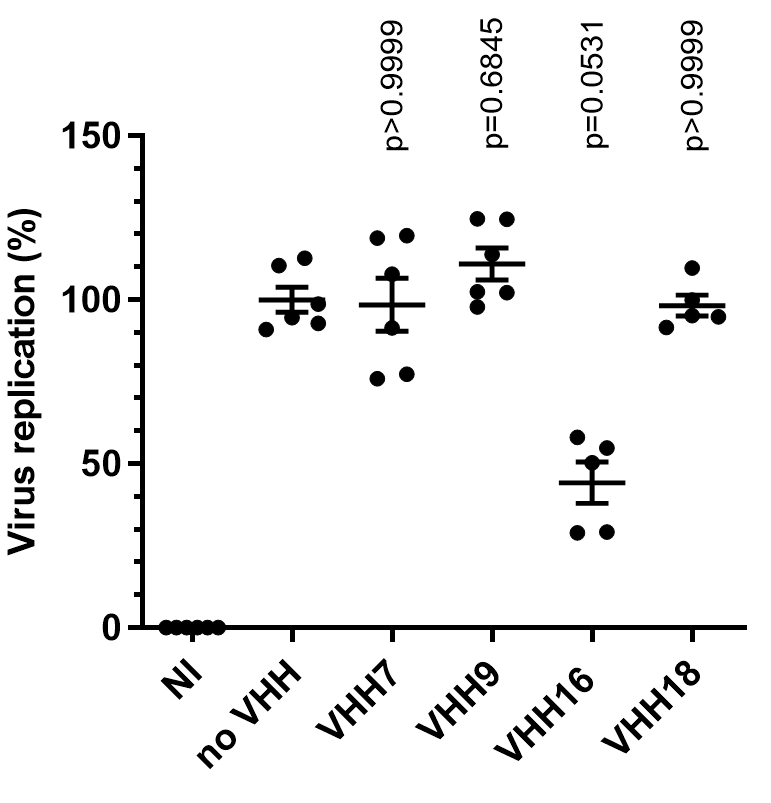

Supplement: S3 Fig — Data are mean ± s.e.m. n = 2 independent transfections with n = 3 technical replicates. Matt-Whitney test was used to compare replication in the presence and absence of VHHs at 24 hours post-infection. (TIF) [file ppat.1011642.s003.TIF]

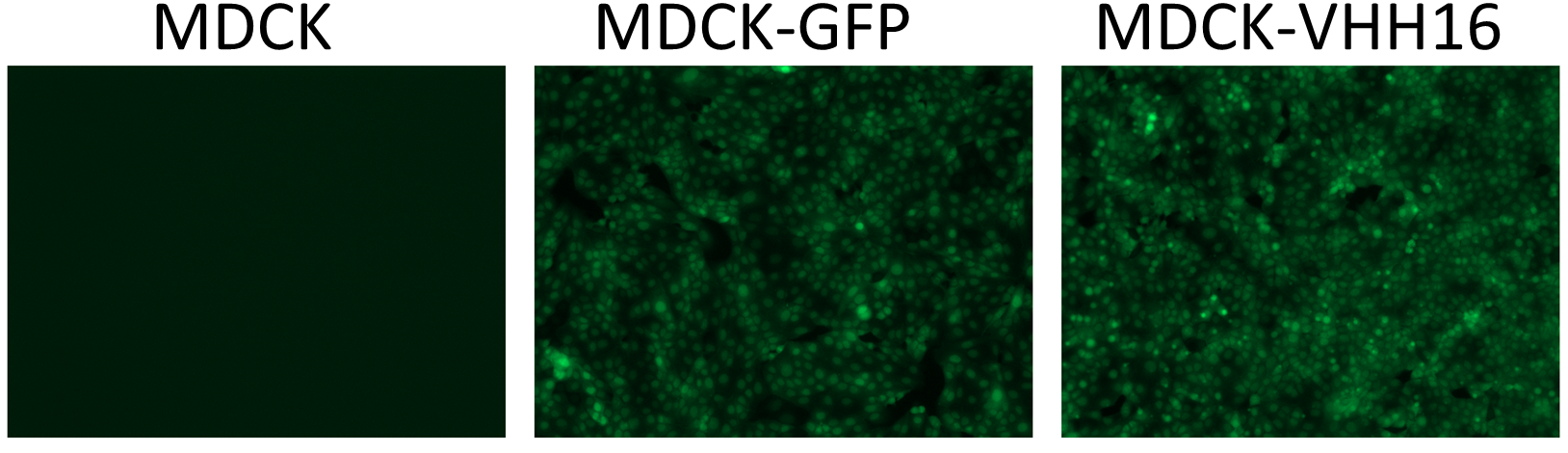

Supplement: S4 Fig — (TIF) [file ppat.1011642.s004.TIF]

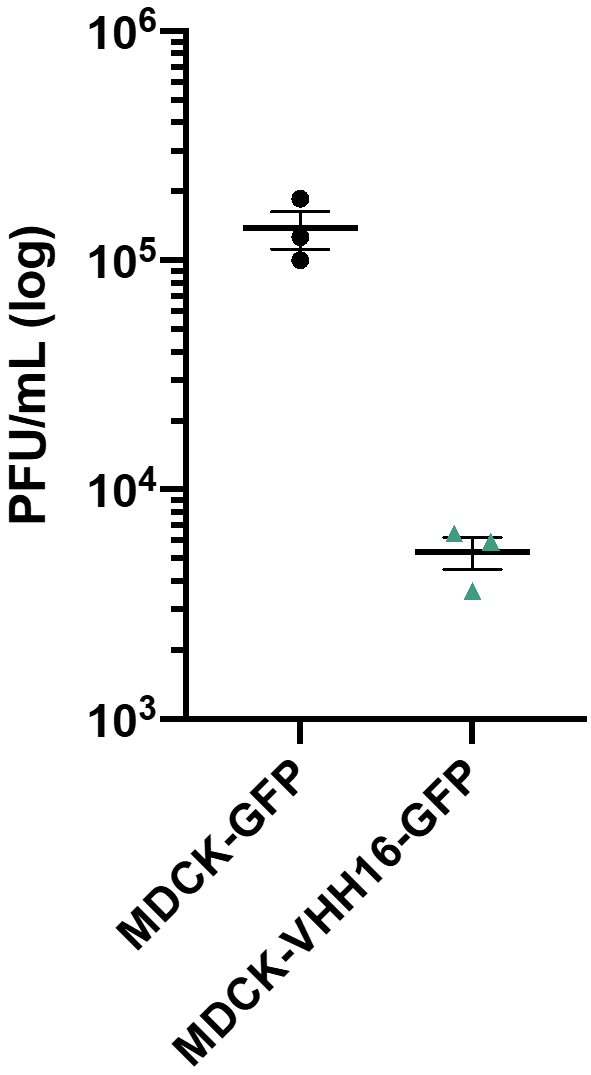

Supplement: S5 Fig — Cells were infected with the H1N1 WSN strain at a multiplicity of infection of 1. Virus production was measured 24 hours post-infection by plaque formation in MDCK cells. (TIF) [file ppat.1011642.s005.TIF]

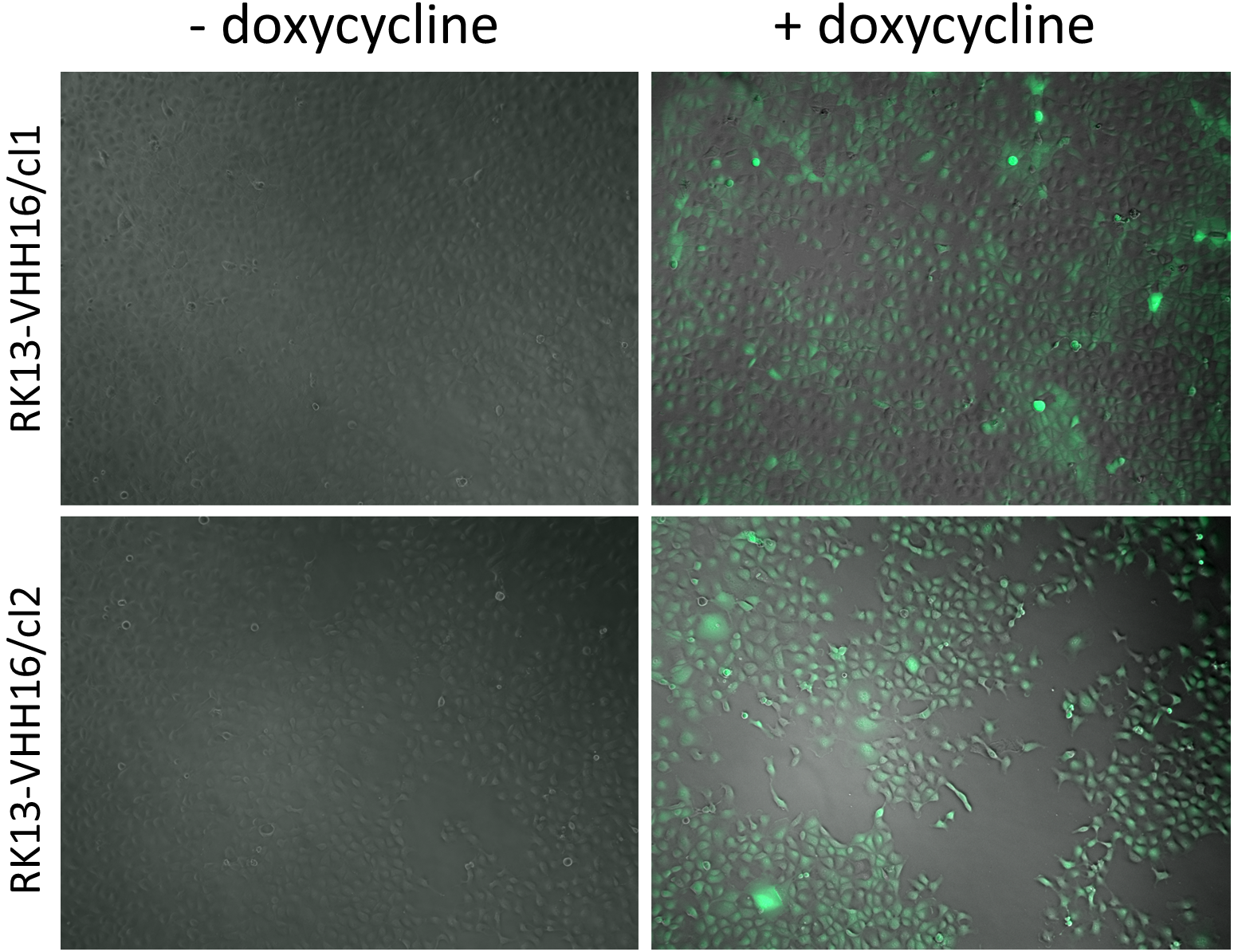

Supplement: S6 Fig — (TIF) [file ppat.1011642.s006.TIF]

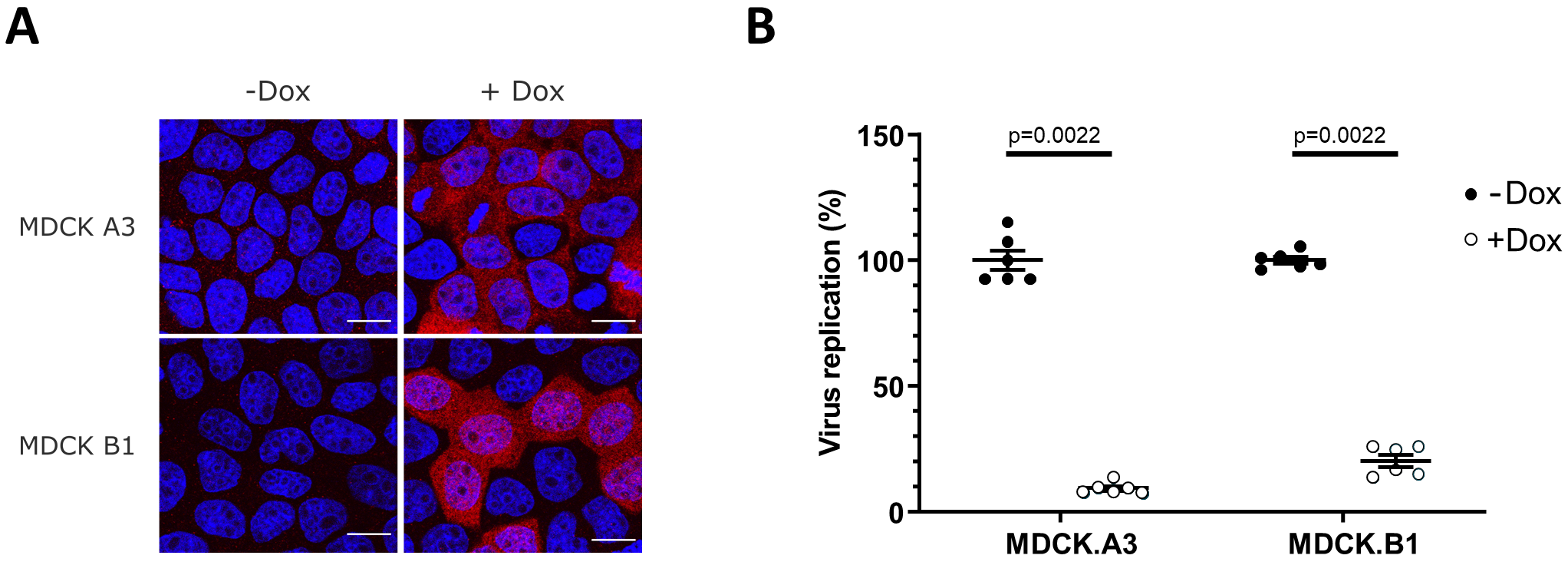

Supplement: S7 Fig — B. Two different MDCK cell clones selected for VHH16-2A-GFP gene expression were incubated (or not) with doxycycline and infected with the reporter influenza virus WSN-Luc. Twenty-four hours post-infection, virus replication was quantified by measurement of the luciferase activity. Data are mean ± s.e.m. n = 2 independent transfections with n = 3 technical replicates. (TIF) [file ppat.1011642.s007.tif]

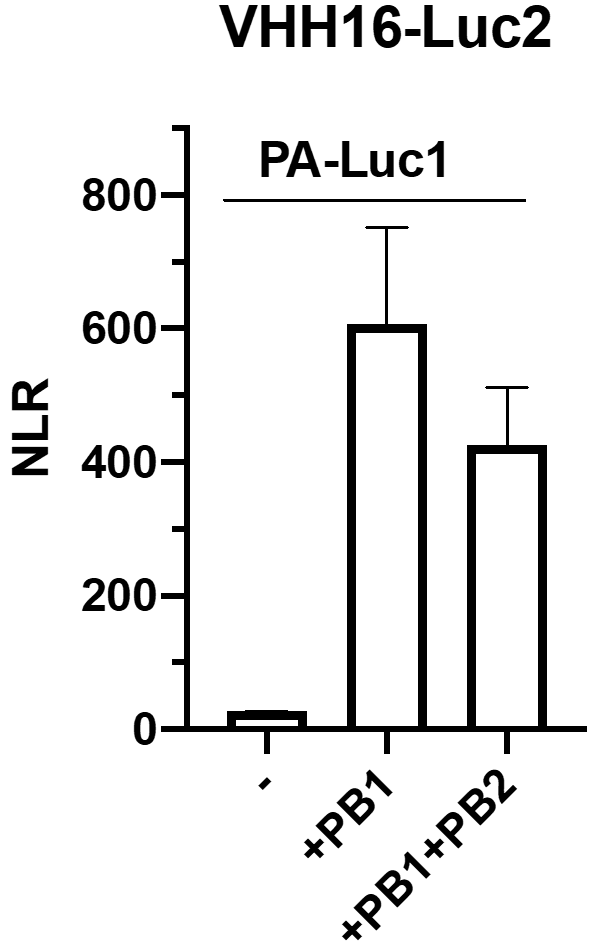

Supplement: S8 Fig — The normalized luminescence ratio (NLR) is calculated as described in the Materials and Methods section to quantify the interaction between VHH16 fused to Luc1 and PA fused to Luc2. VHH16 and PA were expressed with or without PB1 and PB2 subunits. Twenty-four hours post-transfection, cells were lysed and luminescence was measured. Data are mean ± s.e.m. n = 4 technical replicates. (TIF) [file ppat.1011642.s008.TIF]

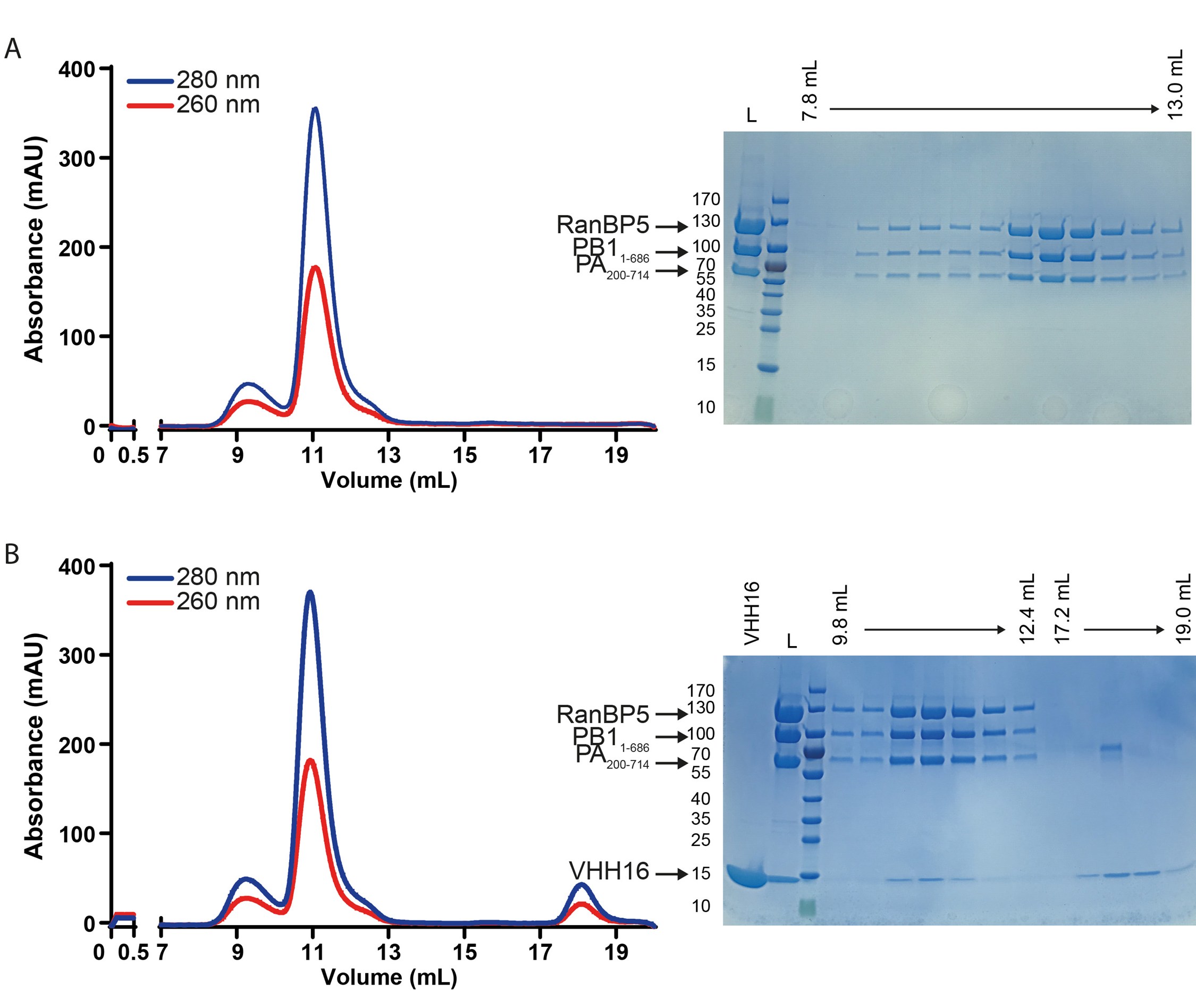

Supplement: S9 Fig — Purified PA200-714-PB11-686-RanBP5 assemblies were incubated with VHH16. Gel filtration chromatograms of PA200-714-PB11-686-RanBP5 (A, left panel) and PA200-714-PB11-686-RanBP5 incubated with VHH16 (B, left panel) were shown. Aliquotes of fractions were analyzed by SDS-PAGE and Coomassie blue staining (right panels). VHH16 was found to stably bind the PA200-714-PB11-686-RanBP5 assemblies. (TIF) [file ppat.1011642.s009.TIF]
