# Supplementary material for: Antiviral activity of intracellular nanobodies targeting the influenza virus RNA-polymerase core
Source: PLoS Pathog. 2024 Jun 14;20(6):e1011642. doi: 10.1371/journal.ppat.1011642 (PMC11210859; doi:10.1371/journal.ppat.1011642)
Supplement: Attachment — Submitted filename: 240229LettreEditeur.pdf [file ppat.1011642.s010.pdf]

RÉPUBLIQUE FRANÇAISE
Liberté
Égalité
Fraternité

UNIVERSITÉ DE VERSAILLES ST-QUENTIN-EN-YVELINES
université PARIS-SACLAY

INRAE

Dr B. Delmas,
Research Director
Coronavirus team
Unité de Virologie et Immunologie Moléculaires (VIM)
INRAE
78350 Jouy-en-Josas
France
Tél. : +33 1 34 65 26 15
Mobile : +33 6 83 23 85 36
Email : bernard.delmas@inrae.fr

To the Editor of *PLOS Pathogens*

Jouy-en-Josas, 29th February, 2024

Ref. : PPATHOGENS-D-23-01477

Dear Editor,

Thank you very much for your return on our manuscript "Antiviral activity of intracellular nanobodies targeting the influenza virus RNA-polymerase core".

Please, find below our response to the reviewers comments and a description of the changes we have made in the manuscript. Following your comments, we attenuated some conclusions we may have overinterpreted without compromising our findings and the interest of our study.

Strinkingly, we improved our understanding of the molecular mechanism of action of VHH16 on influenza virus replication by the identification of its binding site on FluPol. By generating VHH16-escape mutants in VHH16-expressing cells, we have been able to map the amino acids substitutions associated to VHH16 escape. Interestingly, mutations cluster in the PB1 N-terminus and in an adjacent PA α-helix. Using a functional assay of FluPol replication+transcription activity, we validated the importance of this domain, allowing us to define the VHH16 epitope. Importantly, this binding site overlaps with ANP32A and RNA-Pol II binding sites. This structural model combined with our previous data allows us to propose that VHH16 acts on several key functions of FluPol.

Furthermore, we answered the specific questions of the reviewers.

We would like to thank the reviewers for their help to increase the interest of our manuscript. We hope hearing from you soon on the final acceptance of our manuscript.

With our best regards,

B Delmas

RÉPUBLIQUE FRANÇAISE
Liberté
Égalité
Fraternité

UNIVERSITÉ DE VERSAILLES ST-QUENTIN-EN-YVELINES
université PARIS-SACLAY

INRAE

Reviewer's Responses to Questions

Our responses are printed in blue.

Part I - Summary
Please use this section to discuss strengths/weaknesses of study, novelty/significance, general execution and scholarship.

Reviewer #1: In this manuscript, Bessonne and colleagues have generated seven nanobodies against the influenza A virus polymerase using a complex of the PB1 polymerase subunit and the C-terminal domain of the PA polymerase subunit as target. They found that two of these nanobodies inhibited polymerase activity in a minigenome assay, and one of them inhibited influenza virus replication in cell culture. The authors attribute this inhibitory effect to a block in the nuclear import of the PB1-PA dimer.

While the manuscript presents some intriguing data, the authors appear to miss the opportunity to fully capitalize on their initial success in developing nanobodies targeting the influenza virus polymerase. While they convincingly demonstrate that VHH16 effectively inhibits viral replication and the data are consistent with VHH16 impeding the nuclear import of PB1-PA, **the conclusion that VHH16 does not inhibit RanBP5 or PB2 binding is less strongly supported (see specific points)**. The study's impact could be substantially enhanced by **presenting structural models** to reveal where the inhibitory and non-inhibitory nanobodies bind PB1-PA.

We answered to these different points below. Thanks to VHH16-escape mutants and their genomic and functional characterization, we now present a structural model showing that VHH16 binding site overlap or is close to the binding sites of critical host proteins that support influenza replication and transcription, namely ANP32A and RNA-Pol II. We now propose that VHH16 inhibitory activity is mediated by impairing FluPol transport, and blocking both replication and transcription. These data are presented in new figures (8, 9, 10 and 11).

Reviewer #2: In this work the authors identified nanobodies targeting the influenza polymerase from PBMCs of immunized llamas. The authors then tested the most potent nanobody, VHH16, in cell lines for anti-influenza activity. Overall the manuscript is neatly written and experiments were appropriately designed. However, given the level of completeness of the work, **the reviewer struggled to accept the manuscript for publication** in PLOS Pathogens. There are also some concerns on data quality, as I shall elaborate below.

We now increase the completeness of our work by identifying the VHH16 epitope on FluPol.

Reviewer #3: The manuscript by Bessonne et al., investigates the mechanism of inhibition of viral replication by VHH16, a llama nanobody raised against the core of the influenza A/H3N2 polymerase (PA/197-716+PB1+PB2/1-116). Using both the minigenome assay and virus infected cells, they show that intracellularly expressed VHH16 potently inhibits viral RNA synthesis and viral replication (even when induced 6 hours post infection) and this holds for various FluA subtypes (H1N1, H7N1, H3N2) but not FluB. They further show by split luciferase complementation that interacts with PA alone but not PB1 or PB2. However, the interaction with the PA-PB1 heterodimer is much stronger (in the nanomolar range). Using the same assay, VHH16 does not inhibit PB2 assembling with PA-PB1.

However, the nanobody leads to reduced (but not eliminated) nuclear import of the PA-PB1 heterodimer whilst not preventing the interaction with RanBP5, its nuclear import receptor.

The authors do not identify the epitope of VHH16, nor the exact mechanism of replication inhibition (since heterotrimeric polymerase does still form in the nucleus), but suggest that its epitope could be an affective target for antiviral therapy.

The data in the paper is generally convincing and supports the conclusions. However, while it is interesting to have generated such a nanobody, **the manuscript would be much stronger if it included the proposed structural studies** to identify the epitope, which **by cryoEM** should be not too difficult.

We now mapped the VHH16 binding site on the FluPol surface by selecting VHH16 resistant mutants and carrying out their genomic + functional characterizations. This mapping allows us to propose additional modes of VHH16 inhibitory activities.

Part II – Major Issues: Key Experiments Required for Acceptance
Please use this section to detail the key new experiments or modifications of existing experiments that should be absolutelyrequired to validate study conclusions.

Generally, there should be no more than 3 such required experiments or major modifications for a "Major Revision" recommendation. If more than 3 experiments are necessary to validate the study conclusions, then you are encouraged to recommend "Reject".

Reviewer #1: 1. Fig. 3A. The authors should include **Western blot data** to demonstrate that the nanobodies are expressed in HEK-293T cells. The lack of inhibition observed for the remaining five nanobodies could be due to lack/reduced expression. **Nanobody expression should also be demonstrated** for the infection experiment in Fig. S2.

We fully agree that a lack of inhibition by a VHH could be due to a VHH reduced expression (or to an intrinsic stability). We carried out an indirect immunofluorescence assay to validate VHH expression in this transient transfection assay (data shown in a new S2 Fig).

2. Fig. 3D. **The rationale of this experiment is poorly explained**. If the authors' aim was to address whether VHH16 affects transcription or replication or both a more appropriate assay would have been to analyse the effect on primary and secondary transcription and genome replication during viral infection. Instead, the authors use a polymerase mutant supposedly deficient in replication, but **it is not shown whether this mutant is replication-deficient in the experimental setting used here**.

It has been very convincingly shown by the group of Fodor that the FluPol PA-C95A mutant is replication-deficient (Nilsson-Payant et al., 2018, JVI, Fig 2). This was demonstrated using the same experimental set-up than the one we used: a transient transfection assay carried out in HEK-293T cells with the same set of plasmids. We added a sentence in the text to state this fact. To better explain the rationale of the experiment, we added a new experiment in which we quantified by RT-qPCR the amount of mRNA, cRNA and vRNA in infected cells (new Fig 7A). All these data are now described in a new Fig 7. The rationale of these experiments is now better explained.

3. Replicates and statistics. **Numbers of biological and technical replicates need to be specified** (Fig. 4A, Fig. 5C, D, E, Fig. S6) and error bars should be defined (Fig. 4A); please check the calculation of p values in Fig. 3C, D (all set to p=0.028), Fig. 5B, E (p=0.0022) and Fig. 6A (p=0.028). **In Fig. 3A technical replicate** data points from the different transfections **should be distinguished**. In Fig. 6A and 7A specify number of experiments; "representative of several experiments" (lines 513 and 524) is too vague.

We added these information in the figure legends. Fig. 4A: errors bars are defined, but are sometimes difficult to visualize. Fig. 3C and 3D (now in Fig 7B), Fig. 5B and Fig. 6A: we checked again and we found the same values.

We changed the Fig 3A to S1 Fig, and we indicated by a color code the two biological replicates. The legend has been changed, accordingly.

For Fig. 6A and 7A, we specified the number of experiments.

4. Fig. 6A and 7A. **These experiments require Western blots to show the expression levels of polymerase subunits** (both tagged and untagged). Otherwise, the authors cannot conclude that VHH16 preferentially recognises the PB1-PA dimer. The increased value might simply reflect the increased levels of PA expression in the presence of PB1. The discussion around the more efficient binding of VHH16 to the PB1-PA dimer (lines 251-261) is only relevant if the authors show data that the observed enhanced interaction is not due to higher amounts of PA-Luc1 being present. Also, **it would have been potentially informative to include data for interaction between VHH16-Luc2 and PA-Luc1+PB1+PB2** addressing whether PB2 influences nanobody binding.

Fig. 6A. We modified the conclusion of this experiments. Since we now mapped unambiguously the VHH16 binding site on the interface between PA residues and the N-terminus of PB1, we now only state from these data: "suggesting that VHH16 recognizes preferentially the PA-PB1 dimer than the monomeric PA or that PB1 stabilizes the VHH16 binding site on PA" (lines 195 to 197).

Fig. 7A. Our conclusion was that "Co-expression of VHH16 or VHH7 with the three FluPol subunits did not modulate final FluPol assembly". We modified the sentence, to state "Co-expression of VHH16 (or of the non-neutralizing VHH7) with the three FluPol subunits does not result in a significant decrease of the NLR, suggesting that VHH16 does not sterically hinder the PA/PB1 interaction to PB2."

We now included a new supplemental figure showing that PB2 does not interfere with the binding of VHH16 to PA-PB1 (new S8 Fig).

5. Fig. 7D. While this experiment is suggestive that VVH16 binds the PB1-PA-RanBP5 complex, in the absence of further controls it is not conclusive. **Presumably VVH16 is absent from the 9.8-12.4 ml fractions if no PB1-PA-RanBP5 is present but this is not shown**. **Correct FluPol-RanBP5** to PB1-PA-RanBP5 (line 531).

We integrate the corresponding gel filtration to better describe the elution profile and the elution volume of the two binders. We moved the Fig. 7D to a new S9 Fig.

Reviewer #2: (1) In Figure 3, a minireplicon assay was employed to quantify effects of different VHHs. The reviewer finds it uncomfortable to accept the results with the large error in each set.

Besides, the authors should quantify the expression levels of each RNP component and **the co-expressed VHH in each set by Western blotting**. A protein co-expressed at high expression level may (in my experience) sometimes exhaust the cells. Hence, it is necessary to **include a proper control** (an empty vector or, best, a plasmid expressing an irrelevant VHH), which is not seen here.

Fig. 3A is now removed and replaced by data presented in the previous S1 Fig (showing the VHHs inhibitory effects on a H3N2 FluPol) for the following reason: We made the VHH screening with llama immunized with a H3N2 FluPol (as mentioned in ref 20, and in contrast to what we wrote by mistake in page 6, line 104). Now, results are statistically more accurate than previously. We carried out an indirect immunofluorescence assay to validate VHH expression in this transient transfection assay (data shown in a new S2 Fig). A proper control was always integrated in the analyses. "No VHH" means the use of an empty vector. We added this information in the figure legend. We also now better indicated in the different panels the FluPol (H1N1, H3N2 or of the type B) we used in each assay. For the data now presented in S1 Fig (obtained with the H1N1 FluPol), we labeled with two different colors results biological replicates.

All our minigenome data are normalized using a plasmid encoding the nanoluciferase to validate transfection efficiency: this allows us to ensure the lack of cytotoxicity of the VHH. In addition, on the 150 ng of DNA transfection / P96 well, only 10 ng of VHH-encoding plasmids were used.

(2) Figure 5: The authors used a recombinant virus expressing a PA-NLuc fusion as reporter. Theoretically this should work well in most circumstances. However in this particular case it would be difficult to validate the applicability of this virus when the nanobody actually targeted the same PA protein. The authors are suggested **to validate the viral titre with TCID50 assay or standard plaque assay**.

We carried out this standard plaque assay using the wild-type WSN strain and described the result in a new S5 Fig.

(3) The authors did not demonstrate (or at least discuss) clearly the mechanism involved. **The facts that PA-PB1 nuclear import being impaired and PA-PB1-RANBP5 not being interupted seem contradictory**. This follows that the discussion on the mechanism should be rewritten to give more evidence-supported claims instead of putting forward too many guessings (e.g. the sentences about NP look irrelevant).

We previously published a study in which we obtained PA temperature-sensitive mutants that are defective in the PA-PB1 transport to the nucleus, while RanBP5 interacts with a hairpin of PB1 (Da Costa et al., 2015, PMID 25855727) demonstrating no obligatory correlation between RanBP5 binding and PA-PB1 nuclear transport. With the mapping of the VHH16 binding site we carried out (with PA S405, PA Q408 and PB1 D2 defining the epitope), we fully remodeled the discussion section. Sentences about NP have been now deleted.

(4) Related to (3), **Figure 7D is not clearly presented and not stated in the Methods section**. If this is from a gel filtration experiment, the elution profile (chromatogram) should be provided. Besides, when a discrepancy as stated in (3) occurs, it is worthwhile to validate this result in cells (e.g. by co-immunoprecipitation) using full-length PA instead of purified proteins.

We now present Figure 7D in a new clearer S9 Fig that include gel filtration elution profiles. We now describe the experiment in the Methods section. Our objective was to determine if VHH16 is able to

displace the binding of RanBP5 to PA-PB1 dimer, a feature that can be well evidenced by this biochemical study.

PS -- there is a large input band for VHH16 (lane 1). However the eluted VHH bands look weaker than expected. **Was the binding of VHH16 not as efficient in this system as required to displace/block RANBP5**?

As we described now in the Methods section, we made the gel filtration assay with a 2-fold higher concentration of VHH16 compared to $PA_{200-714}$-$PB1_{1-686}$-RanBP5. As shown in Fig. 6, the affinity of VHH16 for the PA-PB1 dimer is below the nM range. Since we observed a partial inhibition of the transport of PA-PB1 into the nucleus in the presence of the VHH, we thus expected that it may be able to displace RanBP5.

(5) There has been reports of nanobodies targeting surface proteins of flu virus (HA/NA) which produced good potencies. These nanobodies can reach the target binding site on cell surface. However few reports nanobodies targeting internal proteins of the virus. All experiments in the manuscript involved the use of transfected plasmids to express the nanobody which is not practically feasible in animal experiments. Hence the authors are recommended to perform in vivo experiments (maybe by infecting mice with AAVs?) to demonstrate that these nanobodies can reach their target proteins in vivo and elicit their effects.

The objective of our study was to identify vulnerability sites on the surface of the FluPol core. We now succeeded with the fine mapping of the epitope of VHH16 at the interface of the N-terminus of PB1 with the PA a-helix [405-415]. Identifying such a vulnerability site in FluPol will help further developments of antivirals for an in vivo delivery, which is not the scope of the present study.

Reviewer #3: **Structural studies should be pursued** to identify the epitope and yield more light on the mechanism of inhibition.

We fully agree that the identification of the VHH16 epitope on FluPol was necessary to better understand the mechanism of inhibition. To this end, we were able to select variants of influenza A virus able to grow in the presence of VHH16 stably expressed in permissive cells. Nucleotide sequencing of their PA and PB1 segments allowed the identification of three contiguous positions at the PB1 N-terminus and the PA a-helix [405-415]. We further validated these data using a minigenome functional assay. Importantly, we were able to show that the VHH16 binding site overlap or is at the vicinity of binding sites of ANP32A and the RNA-polymerase II.

Part III – Minor Issues: Editorial and Data Presentation Modifications
Please use this section for editorial suggestions as well as relatively minor modifications of existing data that would enhance clarity.

Reviewer #1: 6. Fig. 2B. There are no residues white on a red background in the figure (lines 160-161).

Modified in the legend of Fig 2.

7. Fig. 2S. Why only a selection of nanobodies was tested in the virus inhibition assay? Nanobodies

with no detectable effect on polymerase activity could still inhibit virus replication be interfering with a step downstream of viral RNA synthesis and could be of interest. Also, please speculate why VHH18 that was found to inhibit polymerase activity had no impact on viral replication.

Yes, we agree that we could have check the activity of the VHH in a virus inhibition assay. We only focused on the FluPol RNA-polymerase activity, and not downstream steps. Regarding VHH18, we added a sentence to note this point (now lines 146-147).

8. Fig. 5C and line 157. Clarify units of doxycycline concentration (uM in the text vs ug/mL in the figure). Include information on the time of doxycycline treatment and infection.

We corrected to mg/ml.

9. Fig. 7B, C. The description of experimental conditions needs improvement. Which cells were used in this experiment and at what time point was GFP analysed?

HEK-293T cells have been used and GFP signal evaluated at 24 hrs post-transfection. These information have been added in the figure legend.

How many replicates have been performed? The data in panel C seem to represent data from a single experiment. Y axis is not defined.

Two different experiments have been performed. 50 cells were counted for each condition. Y axis is now defined.

10. Lines 244-246. The data show that only two nanobodies inhibited reporter gene expression significantly.

Sorry, we modified to "two" (now line 285)

11. Lines 247-249. Please speculate on how the block of infection at late time points fits with the proposed mode of action of limiting the PB1-PA import into the nucleus. Also, please speculate how VHH16 could inhibit nuclear import without affecting RanBP5 binding.

Having mapped the binding site of VHH16 as the vicinity of ANP32A and RNA-polymerase II binding sites, we now propose that VHH16 delays the import to the nucleus, and blocks replication and/or transcription, as well.

Reviewer #2: Methods: Bio layer --> Biolayer?

Corrected

Reviewer #3: (1) P6, line 104. Here it says that the llama was immunised with WSN core but in the methods and reference 20 it says the construct was the H3N2 core.

We made a mistake and corrected in the text to H3N2 core.

(2) P9, line 182, Figure 6A. Can the authors explain why the NLR with PB1-Luc1+PA was only 10, whereas that with PA-Luc1+PB1 was 175? It should be stated whether Luc1 was on the N- or C-ter of PA and PB1?

Yes, in the split-luciferase system, it is well established that the position of the binding reporters (Luc1 and Luc2) in the complex modulates the NLR signal. Thus you can expect a difference in the NLR signal when you locate your reporters in the N- or the C-terminus of the binding partners. We stated (now line 190) that the luciferase moieties were fused at the C-terminus of the binding partner.

(3) P10, line 212. The split luciferase complementation suggests that PA, PB1 and PB2 can assemble as a heterotrimer in the presence of VHH16. This is not surprising as the antigen used to generate the nanobody included the major stable PB1-PB2 interface (PB1-Cter with PB2-Nter). The experiment does not determine whether the presumed VHH16 bound heterotrimer is functional in binding the vRNA promoter or in RNA synthesis. Does the nanobody bind to the full recombinant trimeric polymerase in vitro?

The complete (and assembled) H3N2 FluPol has never been produced successfully (see Swale et al., 2016, PMID 27095520). Thus, we have not been able to test VHH16 binding on the functional purified FluPol.
