# Supplementary material for: Antiviral activity of intracellular nanobodies targeting the influenza virus RNA-polymerase core
Source: PLoS Pathog. 2024 Jun 14;20(6):e1011642. doi: 10.1371/journal.ppat.1011642 (PMC11210859; doi:10.1371/journal.ppat.1011642)
Supplement: Attachment — Submitted filename: 240502.LettreEditeur.pdf [file ppat.1011642.s011.pdf]

Dr B. Delmas,
Research Director
Unité de Virologie et Immunologie Moléculaires (VIM)
INRAE
78350 Jouy-en-Josas
France
Tél. : +33 1 34 65 26 15
Mobile : +33 6 83 23 85 36
Email : bernard.delmas@inrae.fr

To the Editor of *PLOS Pathogens*

Jouy-en-Josas, May, 02, 2024

Ref. : PPATHOGENS-D-23-01477R1

Dear Editor,

Thank you very much for your positive return on our manuscript entitled "Antiviral activity of intracellular nanobodies targeting the influenza virus RNA-polymerase core".

Please, find below our response to the reviewers comments and a description of the changes we have made in the manuscript (response in blue characters).

Part I - Summary

Reviewer #1: This is a revised version of a manuscript by Bessonne and colleagues considered earlier for publication in PLoS Pathogens. The authors have substantially improved their manuscript. Most importantly, using passaging experiments of influenza A virus in the presence of the inhibitory nanobody and identifying mutants that escape inhibition, they now provide convincing evidence that the nanobody binds at the interface of PA and the N-terminus of PB1.

However, some issues still remain that should be addressed.

Reviewer #2: This is a revised manuscript submitted by Bessonne and co-workers. The authors have enriched the manuscript by providing more mechanistic details of VHH16 action, a nanobody identified by the team to be active in suppressing influenza polymerase activity. As such, the data now better support the conclusion. The data quality has also improved. I recommend acceptance of the manuscript upon the minor revision stated in part III.

Reviewer #3: The revised manuscript has satifactorally addressed/corrected a number of issues raised by the various referees. Furthermore, the revised manuscript is strengthened by identification of the binding site of VHH16 via generation of escape mutants, which enables a plausible explanation of the inhibitory effect to be deduced as it overlaps the Pol II CTD and (presumed, based on the FluC structure) ANP32 binding sites.
On the other hand, the fact that a similar nanobody, with an identical CDR2 binding loop, has already been functionally and structurally characterised (Nb8191, Keown et al, 2022) now considerably detracts from the novelty of this study.

We thank the referees for their careful reading of our revised manuscript. Considering the remark of Reviewer 3, we now discussed the differences and similarities between VHH16 and Nb8191 (see below).

Part II – Major Issues: Key Experiments Required for Acceptance
Please use this section to detail the key new experiments or modifications of existing experiments that should be absolutely required to validate study conclusions.

Generally, there should be no more than 3 such required experiments or major modifications for a "Major Revision" recommendation. If more than 3 experiments are necessary to validate the study conclusions, then you are encouraged to recommend "Reject".

Reviewer #1: None

Reviewer #2: Nil.

Reviewer #3: (No Response)

The authors have no specific answers to address in Part II – Major Issues

---

Part III – Minor Issues: Editorial and Data Presentation Modifications
Please use this section for editorial suggestions as well as relatively minor modifications of existing data that would enhance clarity.

Reviewer #1: Fig. 7B: The authors have failed to address the criticism of their use of the PA-C95A mutant as a replication-deficient mutant without providing any data that this mutant is actually replication deficient in the experimental setting they use. Since this experiment provides little information on whether transcription or replication is affected, I would suggest deleting these data. In the light of the new data it is highly likely that the nanobody inhibits both transcription and replication.

We deleted Fig. 7B as suggested by the reviewer.

Fig. 9C: Please provide the PDB ID for the structure.

Done (PDB number 7NI0)

Fig. S5: Please provide information on the virus used, the MOI, and the timepoint in the legends. What additional information does this figure provide compared to the data in Fig. 4?

The reviewer 3 was concerned by the use of a WSN virus with a reporter gene fused to the PA segment. We thus included a new quantification assay using an unmodified WSN virus in this new

RÉPUBLIQUE FRANÇAISE
Liberté
Égalité
Fraternité

UNIVERSITÉ DE VERSAILLES ST-QUENTIN-EN-YVELINES
université PARIS-SACLAY

INRAE

Fig. S5. As requested by the reviewer 1, we added the information (virus strain, MOI, time point) in the legend.

Fig. S9: The panels and legends lack information; define the blue and red curves, label bands on the gel, and correct the labeling of the polymerase fragments in the legends.

We define the blue and the red curves, we labeled the bands in the gels, and we correct the labeling of the polymerase fragments in the legend.

Reviewer #2: (1) (a) Add, in the Methods section, the relevant experimental details for VHH16 escape mutants generation and the subsequent characterization, presumably by deep sequencing. Parameters for variant calling should be mentioned.

We added a paragraph in the M&M section to describe mutants selection and subsequent sequencing by Sanger DNA sequencing (not deep sequencing) of the isolated mutants. We added a new Table 2 to list the primers we used for RT-PCR amplification of FluPol genes of VHH16-resistant mutants. We added a sentence to specify that we selected 6 mutants that were numbered from 1 to 6.

(b) Why did the authors not validating S405N in their functional validation?

For the functional validation of the site recognized by VHH16, we only needed to show than one substitution alter VHH16 neutralization activity. Since we found that VHH16 promotes substitutions in the PA and the PB1 subunits, we choose to characterize a substitution on each of these subunits.

(2) Briefly discuss the proposed mechanism of action of Nb8191, and explain how it is compared with VHH16.

We added two sentences (lines 334-337) to describe the properties of Nb8191 identified in Keown et al., 2022 (ref 26) and propose that the two VHHs (Nb8191 and VHH16) may have similar biological properties. We added another new sentence (lines 358-360) to state that Nb8191 was able to block Pol II CTD interaction (ref 26).

Reviewer #3: (No Response)

We would like to thank the reviewers for their help to increase the interest of our manuscript. We hope hearing from you soon on the final acceptance of our manuscript.

With our best regards,

B Delmas
